# Promoting Smoke-Free Homes: A Novel Behavioral Intervention Using Real-Time Audio-Visual Feedback on Airborne Particle Levels

**DOI:** 10.1371/journal.pone.0073251

**Published:** 2013-08-23

**Authors:** Neil E. Klepeis, Suzanne C. Hughes, Rufus D. Edwards, Tracy Allen, Michael Johnson, Zohir Chowdhury, Kirk R. Smith, Marie Boman-Davis, John Bellettiere, Melbourne F. Hovell

**Affiliations:** 1 Center for Behavioral Epidemiology and Community Health (C-BEACH), Graduate School of Public Health, SDSU Research Foundation, San Diego State University, San Diego, California, United States of America; 2 Department of Epidemiology, School of Medicine, University of California Irvine, Irvine, California, United States of America; 3 Electronically Monitored Ecosystems, LLC, Berkeley, California, United States of America; 4 Division of Environmental Health, Graduate School of Public Health, San Diego State University, San Diego, California, United States of America; 5 Environmental Health Sciences, School of Public Health, University of California, Berkeley, Berkeley, California, United States of America; 6 Department of Civil and Environmental Engineering, Stanford University, Stanford, California, United States of America; 7 Education, Training, and Research, Inc., Scotts Valley, California, United States of America; Dana-Farber Cancer Institute, United States of America

## Abstract

Interventions are needed to protect the health of children who live with smokers. We pilot-tested a real-time intervention for promoting behavior change in homes that reduces second hand tobacco smoke (SHS) levels. The intervention uses a monitor and feedback system to provide immediate auditory and visual signals triggered at defined thresholds of fine particle concentration. Dynamic graphs of real-time particle levels are also shown on a computer screen. We experimentally evaluated the system, field-tested it in homes with smokers, and conducted focus groups to obtain general opinions. Laboratory tests of the monitor demonstrated SHS sensitivity, stability, precision equivalent to at least 1 µg/m^3^, and low noise. A linear relationship (R^2^ = 0.98) was observed between the monitor and average SHS mass concentrations up to 150 µg/m^3^. Focus groups and interviews with intervention participants showed in-home use to be acceptable and feasible. The intervention was evaluated in 3 homes with combined baseline and intervention periods lasting 9 to 15 full days. Two families modified their behavior by opening windows or doors, smoking outdoors, or smoking less. We observed evidence of lower SHS levels in these homes. The remaining household voiced reluctance to changing their smoking activity and did not exhibit lower SHS levels in main smoking areas or clear behavior change; however, family members expressed receptivity to smoking outdoors. This study established the feasibility of the real-time intervention, laying the groundwork for controlled trials with larger sample sizes. Visual and auditory cues may prompt family members to take immediate action to reduce SHS levels. Dynamic graphs of SHS levels may help families make decisions about specific mitigation approaches.

## Introduction

Second hand tobacco smoke (SHS) is a known human carcinogen and health hazard [1]. It has well-established acute health effects [2] with low thresholds for human sensory irritation [3]. The home remains the major source of second hand tobacco smoke exposure (SHSe) for many nonsmokers, especially children in single-parents households and/or with less-educated parents [4–8].

Previous studies have attempted to reduce children’s SHSe in the home by targeting smoking cessation. However, cessation-based trials have not been consistent in reducing child SHSe [9,10]. Many smokers are unable to achieve or sustain cessation. Thus, interventions that do not rely exclusively on smoking cessation are needed to address SHSe in the home.

Intervention studies focused on SHSe itself have shown some success in using counseling and cotinine or air nicotine feedback to reduce SHSe [10,11]. A recent study using minute-by-minute feedback on airborne particle levels, charts, and motivational interviewing showed reduced SHS levels and changes in smoking-related attitudes [12]. However, until recently studies have used only delayed feedback and imprecise shaping procedures to alter smoking behavior in free living environments, such as private homes, which may have reduced the intervention’s effectiveness. According to the Behavioral Ecological Model (BEM), instant, frequent, and reliable feedback is more powerful in changing behavior than delayed, infrequent, and/or less reliable feedback [13,14]. The theory frames behavior as controlled by consequence contingencies of reinforcement from personal social networks and the community. Emerging real-time behavior change measures and the BEM offer the possibility of new complex and continuous technologies for both shaping behavior and sustaining changes over time, especially with the advent of rich data sets from mobile technologies [15].

Real-time measurement of SHS makes possible a major advance in the delivery of real-time alerts to residents in a smoking home. The feedback informs family and friends who are present, leading to social reactions. With brief instructions to the family, it may be possible to promote responses to the real-time signals that mitigate both current family SHSe and the likelihood of future exposure events.

A workable physical system for providing real-time SHS feedback to households must respond rapidly to smoking events and provide reliable information on SHS levels at intervals of seconds. In addition, to be deployed on a large scale, the components of the system must be affordable. Sensors with the most promise for real-time SHS feedback application are likely to be portable particle measurement devices. Particles are generated in quantity when tobacco is smoked; a cigarette emits roughly 1.4 mg per minute into the air [16].

The concentration of fine particles with diameters smaller than 2.5 microns (PM_2.5_) is commonly used as an indicator of the presence of SHS [17]; the outdoor level of PM_2.5_ is one of the *criteria pollutants* regulated by U.S. EPA and associated directly with health effects [18–20]. A disadvantage of using PM_2.5_ for feedback is that particle devices also respond to non-tobacco aerosol sources, including cooking and incense. Particle feedback is not tobacco-specific, which may compromise the shaping process and complicate data analysis. The near real-time detection of volatile or semi-volatile organic chemical (VOC/SVOC) SHS components for SHS feedback does not appear to be currently feasible. At present, reliable, affordable, and standardized continuous and direct-read devices for tobacco-specific chemical species, such as nicotine, are not readily available.

Many real-time particle measurement devices based on light-scattering are available commercially and can be calibrated to give mass concentrations for different aerosols [21–23]. However, most are priced out of the range of consumers. The challenge for behavioral feedback applications is identification of an inexpensive device that is reliable and sufficiently accurate to consistently and quickly respond to peak particle concentrations due to smoking events in the home -- and with enough resolution to distinguish low and high particle concentrations. Ott et al. [24] report that fine particle levels reached a peak of ~300 µg/m^3^ in a medium-sized 43 m^3^ bedroom after a cigarette was smoked. A model-based analysis by Klepeis and Nazaroff [25,26] predicts particle concentrations in smoking homes reaching peak levels of >100 µg/m^3^ in rooms with an active smoker present and over 40 to 50 µg/m^3^ in hallways and other rooms, depending on a home’s layout and airflow characteristics. Thus, a suitable particle monitor should resolve background concentrations of ~10 µg/m^3^ against peak levels that may exceed 100 µg/m^3^ in order to signal likely smoking events in the same room.

In this study, we developed a prototype real-time SHS monitor and feedback system using a commercially-available particle counter intended for household consumer use. With custom programming and off-the-shelf electronic components, we designed the system with auditory and visual cues, and animated graphics, to trigger instantaneous recognition of elevated SHS particle levels that are likely due to smoking, and, thereby, promote specific immediate SHS mitigation activities. Using the BEM as a guide, we hypothesized that real-time SHS feedback would generate discussion among family members and lead to SHS mitigation attempts, smoking restrictions, home smoking bans, or cessation due to the immediacy of information regarding SHS levels in the home and the direct link between observed levels and reactions from the smoker’s personal network. Our specific goals were to test the feasibility and potential effectiveness of the system by determining: (1) The performance of the system in providing consistent and understandable feedback; (2) The practicality of placing the system in homes for weeks or longer; (3) The opinion and acceptance of the monitor and feedback system in smoking households with children; and (4) The potential for the monitoring system to promote behavior that reduces SHS levels or establishes home smoking bans.

## Methods

### Ethics Statement

The San Diego State University (SDSU) Institutional Research Board (IRB) approved this research on May 8, 2009, stating: "The referenced protocol [for this study] was reviewed and approved in accordance with SDSU’s Assurance and federal requirements pertaining to human subjects protections within the Code of Federal Regulations (45 CFR 46; 21 CFR 50)." A written informed-consent form, approved by the IRB, was signed by all admitted study participants. For participating families with children, the consent form was signed by a parent.

### Development of the Monitoring and Feedback System

We chose the commercially-available Dylos DC1100 particle counter with integrated RS232 PC interface ($290; Dylos Products, Inc., Riverside, CA, USA.) to provide core SHS-sensing capability. This model was targeted at consumer use and was inexpensive enough to allow for wide deployment in a scaled intervention, and appeared adequate for quantifying tobacco smoke levels in homes. We obtained customized units from the manufacturer that enabled fine control of communications via a RS232 port and 4 particle size bins (0.5, 1.0, 1.7, and 2.5 µm). The extra size cuts were not used in our study, but they may enable determination of a source aerosol’s particle size distribution and may be useful for aerosol source identification in future studies.

**Figure 1:**
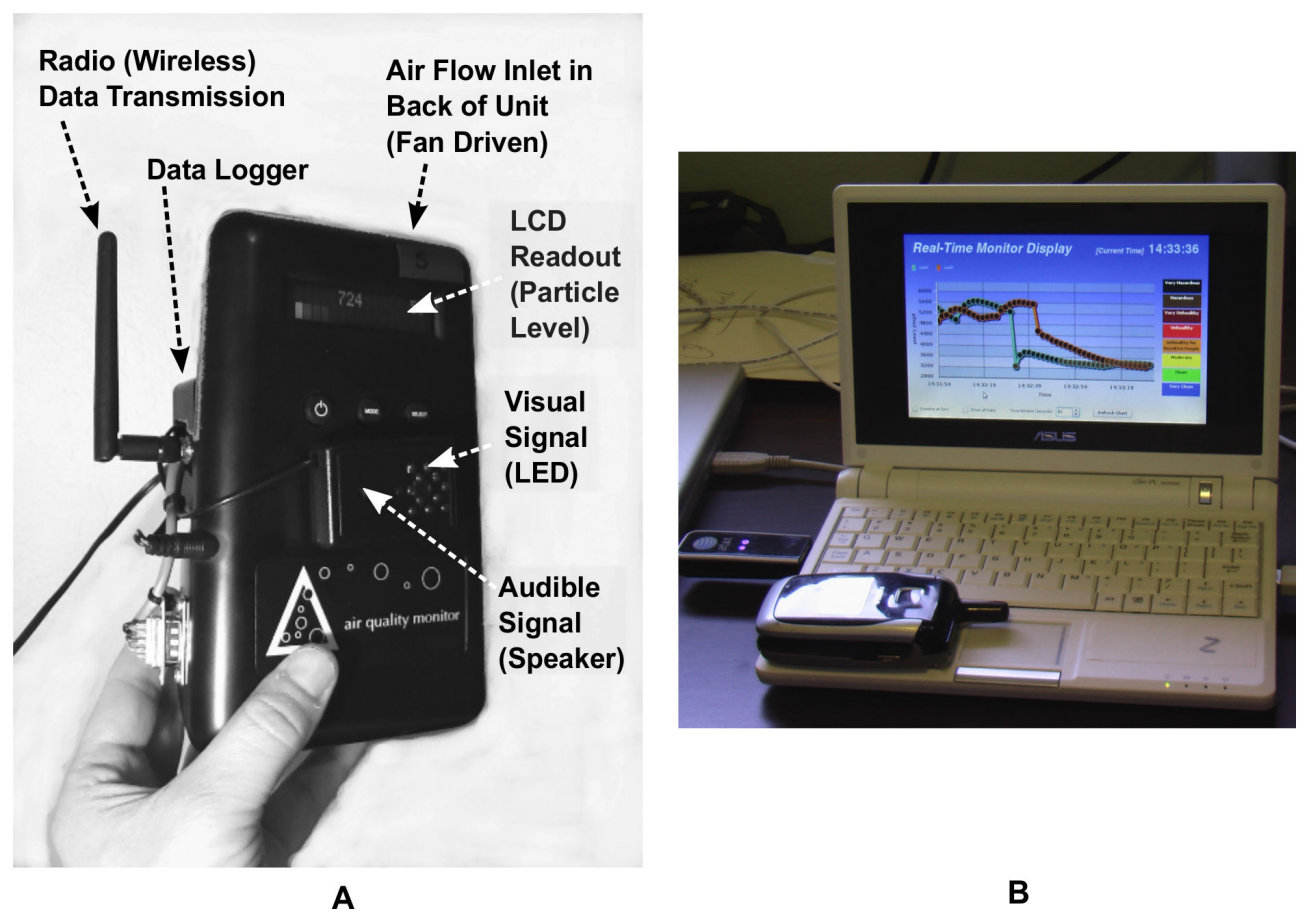
The airborne particle monitor and feedback system with graphical display. **A**. A Dylos particle counter was augmented with a wireless transceiver, visual and audible signals, and additional data storage. **B**. Real-time data on particle levels were transmitted from the customized Dylos to a local *netbook* computer running the Ubuntu Linux or Windows XP operating system, where they were displayed on an animated graph and automatically uploaded via USB cell modem to a remote web-server (in figure, see cell phone placed on netbook keyboard to show scale).

We modified the base Dylos monitor obtained from the manufacturer with an add-on module providing a digital data-logging and communications chip (OWL, EME Systems, Berkeley, CA), a wireless transceiver chip (Digi International Inc., XBee 802.15.4, 2.4GHz, 1mW), and an auditory and visual signaling package (Figure 1A). The system automatically stored time-stamped particle data to internal memory every minute (3-week storage limit) and wirelessly transmitted 10-second readings to a local computer (Figure 1B).

The system firmware was programmed with features for activating auditory and visual cues (beeps and/or flashing lights), defining two variable mass-concentration thresholds at which signals were triggered (particle counts were internally converted to units of µg/m^3^ using coefficients for tobacco aerosols). The auditory signals consisted of a computer-controlled speaker, which was programmed to emit a short beep when a pre-defined mass concentration threshold was exceeded. The visual signals consisted of a grid of computer-controlled LED’s, which was programmed to display a steady-green light during clean episodes (below first threshold), a larger pattern of flashing yellow/orange light when the first threshold was exceeded, and a large “X” pattern of flashing red lights when the second threshold was exceeded.

We developed a secondary feedback mechanism that consisted of real-time mass concentrations displayed as a dynamic graph on a nearby netbook computer with wireless host capability (Figure 1B). We developed both server and graphical user interface (GUI) software using the Perl programming language and animation tools (Flash™, Adobe Inc.), respectively, to process incoming records of particle data and display them in an animated time series chart. To provide an approximate indication of the severity of particle levels, the moving chart was color-coded according to the U.S. EPA, Air Quality Index (ranging from green = clean to maroon = hazardous; http://www.airnow.gov). The server software transmitted data records to a web server via a USB cell-phone modem to facilitate easy data retrieval and data quality checks.

### Laboratory Evaluation

The goal of our laboratory evaluation was to standardize the response of the Dylos particle monitors to PM_2.5_ mass concentration units of micrograms per meter cubed (µg/m^3^), which are commonly used to characterize inhalable particle levels. This procedure serves to establish consistency between individual Dylos units and other particle measurement devices, to allow for comparisons to health standards or PM_2.5_ observed in other settings, and to provide feedback parameter values (e.g., thresholds) that can be easily interpreted and adapted by other researchers regardless of monitor type.

We performed tests of the Dylos particle monitor using a smooth hardboard chamber (55 x 55 x 55 cm) to minimize electrostatic effects on aerosols. A set of 4 aerosol concentrations from cigarette smoking was introduced in discrete ~20 minute periods to the chamber via a 3.8 cm diameter duct from a separate combustion box A small mixing fan was used within the chamber to mitigate any spatial variation of particle concentration. The chamber was flushed between exposure periods with HEPA-filtered particle free air to ensure proper response relative to baseline signal. Limits of detection for each aerosol were also determined, defined as 3 times the standard deviation of the instrument’s signal for a flushed (near-zero) particle environment.

For mass-calibration and evaluation of the Dylos, several established particle measurement devices were used, including a Tapered Element Oscillating Microbalance (TEOM) (Thermoelectron, USA) and a DustTrak (TSI, Inc., Shoreview MN). The TEOM continuously measures particulate concentrations via changes in frequency of an oscillating collection filter at the end of a heated element, and serves as a reference particle instrument. The TEOM response is not affected by the size distribution or optical properties of the test aerosol. The DustTrak is an established and widely-used light-scattering particle monitor in air pollution studies [21,22,27,28] that was used for comparison purposes and to evaluate the overall validity of the Dylos readings. Data from the experiments were averaged during each active-source period to derive mass-conversion coefficients for the Dylos monitor (i.e., a factor for converting instrument response to mass concentrations for the different particle sources). The TEOM was used to calibrate the DustTrak to the mass of tobacco smoke aerosols in the chamber. Subsequently the DustTrak, which had similar baseline and noise characteristics as the Dylos, was used to mass-calibrate each Dylos unit.

### Focus Groups

We conducted 3 focus groups to determine the acceptability of the proposed intervention for households in which smoking occurs and young children may be present. These households were likely to be low-income families. We used purposive (non-random) sampling to select household members for study enrollment and sought their reactions to the monitoring technology and the feasibility of placing the instruments in their homes. Participants were recruited from a short self-administered screening questionnaire placed with the San Diego State University (SDSU) Special Supplemental Nutrition Program for Women, Infants and Children (WIC) sites. The WIC sites serve low to moderate-income pregnant, breastfeeding and postpartum women, and infants/children up to age 5 who are at nutritional risk. Completed questionnaires were placed in a $50 raffle drawing.

Thirteen participants (6 men and 7 women) were recruited to form three focus groups with 3-6 persons per group. The focus groups consisted of nonsmokers and smokers who lived in a household in San Diego County containing at least one smoker. Two focus groups were conducted in English and one in Spanish. Participants’ mean age was 36 years (ranging from 18–52). All had completed high school with half having completed some college. Fifty-four percent were married. Most were current smokers (77%). Participating nonsmokers lived with a smoker. Seventy-seven percent of participants had a child living with them.

Each participant received a snack plus a $45 monetary incentive to cover expenses. The focus groups lasted two hours, on average, and were led by a trained bilingual moderator. The moderator followed a semi-structured discussion guide, beginning with warm-up discussion on the group’s experience and opinion on using “smoke-detector” technology in homes, and followed by a demonstration of the monitoring package (including pictures of it in a home, of the monitor alerts, and of the laptop display showing example particle level increases). The package used for illustration in the focus groups differed in precise components versus the one used in the field tests. Next, the moderator prompted reactions to and suggestions for the monitoring package (e.g., lights, sounds, appearance, tamper-proof, placement, additional features needed). The moderator asked about the acceptability of using monitors to promote smoke-free homes, the potential effectiveness of the monitors, and the types of information displayed on the laptop. Lastly, the group discussed who might benefit, who might be willing to use it, and potential barriers.

All sessions were audio-taped. The transcripts from the individual focus groups were merged into one master transcript; the answers to the same question in each focus group were grouped together and analyzed across the three focus groups for patterns, trends, or themes. The transcripts from the tapes were reviewed by two researchers who discussed discrepancies until agreement was reached.

### Intervention Recruitment and Procedures

The intervention was field-tested in the apartments of three families (denoted F1, F2, F3) located in the San Diego, California (USA) metropolitan area between December 2009 and April 2010. Families were recruited through focus group contacts and WIC centers through drawings that asked questions on health. We enrolled families living in apartments with one or more smokers and a child, or with one or more smokers and a pregnant woman. At the first visit to each home, after explaining that the monitor detects air particles such as smoke and obtaining informed consent, we placed one air monitor in an open living, dining, or kitchen area. A second monitor was placed in a bedroom where a child or pregnant woman slept. A small netbook computer was placed on a table, shelf or counter, where occupants could view the levels and it was out of the reach of children and pets. The netbook received wireless signals from each monitor, displayed them on a real-time graph, and transmitted data to a remote server via a cell phone connection (see description above). During the baseline period (1-week target duration), we configured the monitors to measure the air quality of the home silently (i.e., light and sound signals were not activated). Participants were instructed to behave normally and to ignore the monitors and the netbook. Tape was placed over the LCD display on the monitors and a piece of paper was taped over the computer screen. We visited the home at the end of the baseline measurement period (*post-baseline session*) at which time we showed each participant plots of monitoring time series data from the results of the baseline period (see example plots in Results section below). The charts were used to illustrate to participants key characteristics of SHS:

 Particle levels from smoking create sharp identifiable peaks in the room where smoking occurs

 Particles can persist for long periods in the home in the absence of ventilation

 Particles can spread easily and quickly to other rooms where doors are open

 There are different possible approaches to SHS mitigation that include opening windows or doors, smoking near open windows or doors, smoking near exhaust fans, or smoking outdoors.

After discussing the charts and possible mitigation strategies in a brief coaching session, we explained the operation of the audible and visual signals and the real-time feedback chart to the participants. We did not expressly recommend specific actions, but informed participants regarding possible mitigation responses to the signals and real-time graphical feedback (described above). To launch the intervention, we activated the signals on the monitoring devices for a target intervention period of 1 week. Participants were told that monitor signals would warn occupants when the levels are high so they could take immediate action to reduce SHSe. During the intervention, the tape was removed from the netbook display so participants could view dynamic levels and try various approaches to reduce the particle levels shown on the screen and, thereby, improve the air quality of their home.

During the baseline and the intervention periods, we asked participants to complete a daily diary to record the time when different aerosols were present in the home. We instructed residents to note the timing of smoking events, household ventilation behaviors, and activities generating non-tobacco particle emissions, including cooking, cleaning, wood burning, incense, outdoor grilling, candles, vehicle emissions, woodworking, renovation, vaporizer, consumer aerosol products, or industrial work. We gave residents a list with examples of all non-tobacco sources that should be noted. The time-diary was intended to provide data concerning the identity and type of aerosol that might be detected by the monitor.

Adult smoking and non-smoking household members were interviewed using a structured questionnaire at the start of the baseline period (*baseline interview*) and after the intervention period (*post-intervention interview*) to measure changes in behavior and attitudes regarding SHS. We attempted to perform the interviews with all adult household members present, although sometimes work schedules interfered. We took notes on open discussions that took place in the post-baseline session that occurred before the intervention feedback signals were activated. The baseline questionnaire included questions about the physical characteristics of the home (age, ventilation system, type and number of rooms), number of smoking and nonsmoking occupants, opening of interior doors and exterior windows, timing of smoking activity, location of smoking, current smoke mitigation activities (open windows, move to another room, smoker segregation, smoker goes outside, turn on central air), cooking activities and their timing, cleaning and dusting activities, and the presence of carpeting. The post-intervention questionnaire included questions about opinions and performance of the monitoring system, as well as changes in behavior (Table 1).

**Table 1:** The Post-Intervention Questionnaire.

**No.**	**Question**
1.	Did you hear or see the alarms sound on any days? (never/none)
2.	On which days and general times of day did they sound?
3.	Were you able to hear or see the alarms easily?
4.	Is there anything about the alarms that you think should be changed?
5.	What are your general opinions about the monitors and alarms?
6.	What did you do when the alarms sounded? For example, did you stop smoking, or open windows or doors? (prompt several times to get complete answers)
7.	Did you make a lasting change in your behavior in response to the alarms?
8.	How much do you think the alarms helped to change your behavior versus just knowledge of the data we showed you last time?
9.	Look at the peaks on the chart that correspond to times of smoking, cooking, or other activities you reported on your time diary? Look how peak levels change based on different circumstances in the house. What do you think about the levels shown?
10.	Looking at the chart data, and thinking about how the alarms sounded in different rooms, what would you consider doing further to reduce smoke levels?
11.	Do you think the alarm feedback would cause you to make lasting changes in your smoking or other behavior?

### Data Analysis

To establish the impact of the monitoring feedback on intervention participants’ behavior, and attitudes and opinions of respondents, we analyzed qualitative results from all family interviews for the baseline versus the intervention period. We also present qualitative results from the focus groups. To provide quantitative evidence of the impact of the intervention on SHS levels, we analyzed the before-and-after-intervention 1-minute particle levels in each home, presenting time series plots and descriptive statistics and broad results from the time-diaries. We explored descriptive metrics of the real-time data for potential use in future studies, using the median as a metric of central tendency, since it is a better representation of the data for non-normal (e.g., skewed) distributions and less sensitive to outliers. Using 1-minute aggregated time series data, we studied continuous *episodes* (i.e., time segments) in which levels exceeded the first feedback signal threshold – computing the median of peak levels, average levels, daily number of episodes, daily duration of episodes, and integrated (area-under-the-curve) concentration values. The threshold-based episodic analysis isolates probable smoking events from those unlikely to be associated with smoking, e.g., from background or ambient sources. The integrated levels take into account both particle level and duration of levels, and therefore, are likely to be a preferred metric for estimating changes in occupants’ SHSe.

We wrote software in the R statistical programming environment [29] to perform the time segment analysis described above. The analysis of lab calibration data for the Dylos monitors was performed with R or spreadsheet software.

## Results

### Monitor’s Response to Secondhand Smoke Aerosol in Controlled Experiments

**Figure 2:**
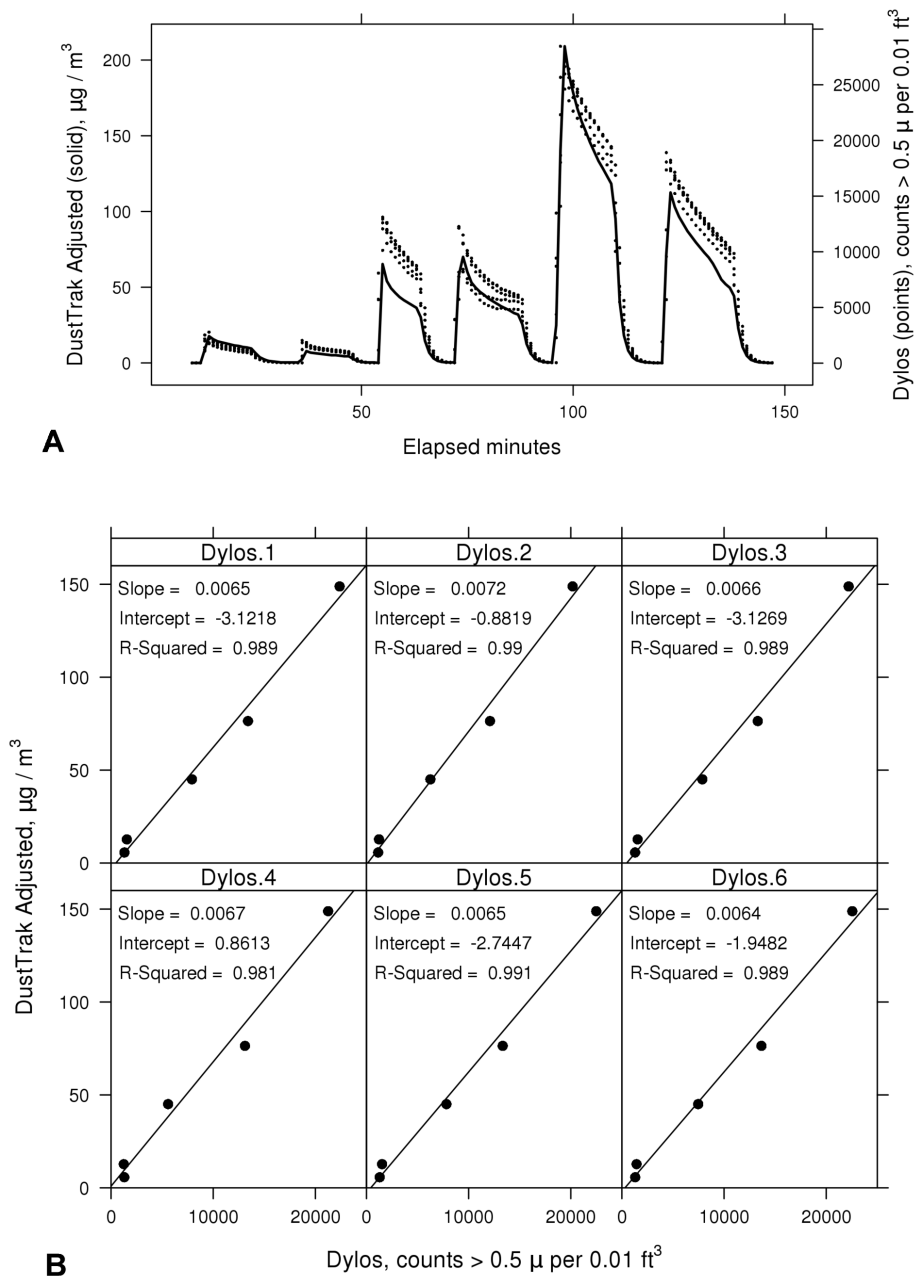
Response of Dylos particle counter versus mass-adjusted DustTrak monitor. **A**. Time series plots of 1-minute readings from six Dylos units used in the present study (points) versus a mass-adjusted DustTrak for a series of 6 cigarette experiments (smooth line). **B**. Linear regressions showing relationship between average Dylos particle counts above 0.5 microns and DustTrak mass concentrations in μg/m^3^ for the six units. Counts above 2.5 microns were negligible.

Based on our laboratory cigarette experiments, we found the Dylos particle counter had comparable sensitivity, precision, and baseline stability compared to the industry-standard DustTrak aerosol monitor (Figure 2A). Like the DustTrak, the Dylos readings were highly-resolved, giving a smooth signal. The Dylos demonstrated little or no drift during near-zero particle environments and did not appear to be impacted by temperature or other environmental conditions. Limits of detection (LOD), defined as 3 times the standard deviation of the instrument signal during a near-zero particle environment, indicate the nominal Dylos mass-concentration LOD is similar to the DustTrak, both 1 µg/m^3^ or lower.

The DustTrak was mass-adjusted (calibrated) against the TEOM instrument, which provided a gravimetric-based particle mass standard. Correlations between the Dylos particle counts above 0.5 microns and the mass-adjusted DustTrak showed a linear relationship spanning 10 to 150 µg/m^3^ with R^2^ values greater than 0.98 and near-zero y-intercepts (Figure 2B). In the lab experiments, since ambient air was HEPA-filtered, the background levels were very low and counts of particles larger than 2.5 microns in diameter (regime for dust) were negligible (i.e., below 1% during source periods). The approximately-linear Dylos response for the cigarette-source experiments supported the use of regression slopes as mass-conversion coefficients for each of the six Dylos units (ranging from 0.0052 to 0.0072 µg/m^3^/response). These coefficients were programmed into the feedback system so that each Dylos raw response value (particle counts per 0.01 cubic feet between 0.5 and 2.5 microns) was automatically multiplied by the mass-conversion coefficient to output a response value in estimated mass-concentration units (µg/m^3^). Our laboratory analysis of the Dylos demonstrated a capacity to measure particle concentrations that are expected to occur in smoking homes -- from low levels in the 10’s of µg/m^3^ to 150 µg/m^3^ or more. Thus, the threshold-based signals for the intervention, which we anticipated to be in the range of 50 to 150 µg/m^3^, could be triggered with reasonable accuracy for SHS-related events using mass-concentration units. The Dylos particle size range between 0.5 and 2.5 microns is in the range of particle sizes associated with SHSe, which are under 2.5 microns in diameter [30]. Particles above 2.5 microns are typically emitted from mechanical processes such as sweeping or vacuuming. Therefore, using feedback triggered based only on particles under 2.5 microns in size provides a way to broadly filter feedback for some non-tobacco sources.

### Social Acceptability of Putting the Monitoring and Feedback System in Homes

The focus groups provided valuable information about the acceptability of using the monitors in homes. Table 2 summarizes typical verbatim sentiments from focus group participants. The participants were positive about the concept of air monitoring in their home and were interested in the monitoring devices to learn about their home’s air quality. They indicated they would be willing to participate in a program using visual and auditory alerts in their homes, for several months or even a year, and that they thought the monitors could be conveniently located in a bedroom or living room. They liked the feedback provided by the lights and sounds from the monitor to alert them of the levels in their homes. They approved of the lights’ colors for the visible signals (green = clean; orange/yellow = warning; red = danger), but wanted to be sure that the audible signal was not too intrusive, although loud enough to be heard, and wanted control over sustained sounds. Some participants suggested having a progressive signal that increased in intensity. Participants were interested in knowing the exact levels of pollution in their home and seeing the level, as well as receiving tips on their meaning and how to respond to them (e.g., use fans or open windows, what times of day are bad, whether individual rooms or the entire home have high pollutants). Most participants were receptive to the idea of receiving tailored information about their home, either via email or telephone. They were also receptive to receiving information via a computer or from a physician.

**Table 2:** Focus Group Discussion Points and Representative Sentiments.

Feasibility Questions and Responses
How do you feel about using monitors to keep homes smoke free?
It can change the way you live…
You think twice before you light a cigarette.
Good knowing your air quality, especially when you have children.
I’m pretty excited about it.
How long could we place the monitors in homes as part of research?
I think a 6 month period.
I think 6 months to a year.
As long as it would take.
Feedback Mechanism Questions and Responses
What do you think about lights and sound?
We all know green is good, yellow is warning and red is danger.
. not like a smoke detector that’s loud, obnoxious and continuous. You don’t want the same type of alarm as a smoke detector, because you wouldn’t want families to say “that’s the air monitor”.
If something bad was in the air, it could be louder, maybe at a moderate pace?
What do you think about showing the particle levels on a laptop screen?
It’s very good to see what the levels of contamination are in the house and the difference we can make. Or you have a message on the computer of the levels and perhaps a tip. It would also be good knowing which room the pollutants are in or what is happening in the whole house.
Others’ Reactions Questions and Responses
What positive or negative reactions do you think other people might have to the feedback?
Lots of people like me don’t want to smoke. I want to quit very badly. Sometimes you just need that extra push in front of your face.
It’s an opportunity to educate people. I don’t think my friends that are smokers would react negatively. If there is something beeping, I’m going to explain. It’s a way of opening up a dialog.
The fact that you have something concrete to show you’re improving is a motivator.
I’d have a hard time sitting there smoking when I realize how much I’m polluting.
If I enter a home that had the monitor and they were non-smokers and they say ‘okay, because you smoked in the house this is what happened’, I would make changes obviously.
Participation Questions and Responses
Who might want to use the monitoring system?
… people who want to quit smoking…
… this is great when you have children or even before.
Participation Barriers Questions and Responses
… the smoker is so hard core they’re just not going to change.
… they’re criticized enough as a smoker… being a smoker is like being a junkie right now. There is very negative connection so approaching them about it could be difficult
Participant Recruitment - Suggestions
Participants suggested that we recruit families that have children with allergies and that we recruit from health clinics, employee lounges, grocery stores and schools.

Analysis of post-intervention questionnaire responses from the 3 intervention participants indicated that the physical features of the monitoring system were acceptable. They all liked the simple design of the monitors’ lights and beeps, and found the colors of the lights to be intuitive, and not annoying. They were able to see and hear the alerts every day, although one participant suggested musical tones and more visible lights with a single LED instead of multiple LEDs.

### Field Performance of the Monitor

Using monitoring data and time-activity responses from the three participating homes, in which the monitoring system was installed, we evaluated the performance of the Dylos monitors using more than 30 full days of continuous readings. We found the monitors to be reliable, although occasionally a monitor was unplugged, wireless communication was lost with the netbook, or the internet connection was lost. Following communication errors, data were usually recoverable from the on-board monitor storage, except for a few days at the end of the intervention in participant home F1.

Based on visual analysis of the baseline data on peak particle levels and time-activities in each home, we selected a first threshold for feedback signals of 60 µg/m^3^, which appeared to capture all smoking events but was not triggered by nearly all background (ambient) levels. We selected a second threshold of 100 µg/m^3^ to provide a secondary signal on rising intensity of the smoke that falls within the range of our Dylos mass-calibration. We found that the background levels, in the absence of apparent indoor sources, were low and below a nominal mass concentration level of 10 µg/m^3^. The 1-minute instrument noise amplitude was approximately 1 µg/m^3^ or less, which is many times lower than the instrument response due to cigarettes or other aerosol-producing activities (e.g., marijuana smoking or cooking). There was no apparent drift in the baseline readings for the instrument. The monitor was sensitive to smoking activity occurring in the same room -- capturing peak events at nominal levels up to 300 µg/m^3^ above background. However, since the monitor was calibrated for data below approximately 150 µg/m^3^, which encompasses both signal thresholds of 60 and 100 µg/m^3^, more uncertainty may be present in the observed absolute mass concentrations above this level. The monitor captured smoking events occurring in a separate room, e.g., measurements taken in a bedroom with smoking activity in the living room, with levels 10 to 40 µg/m^3^ above background. Since the Dylos’ response time is fast (under 10 seconds) and it has a mass-concentration measurement resolution of approximately 1 µg/m^3^ or less, it can potentially be used for precise analysis of the growth and decay of particle concentrations for smoking-associated features in the time series.

### Evidence of Behavior Change in Response to Feedback Signals

**Figure 3:**
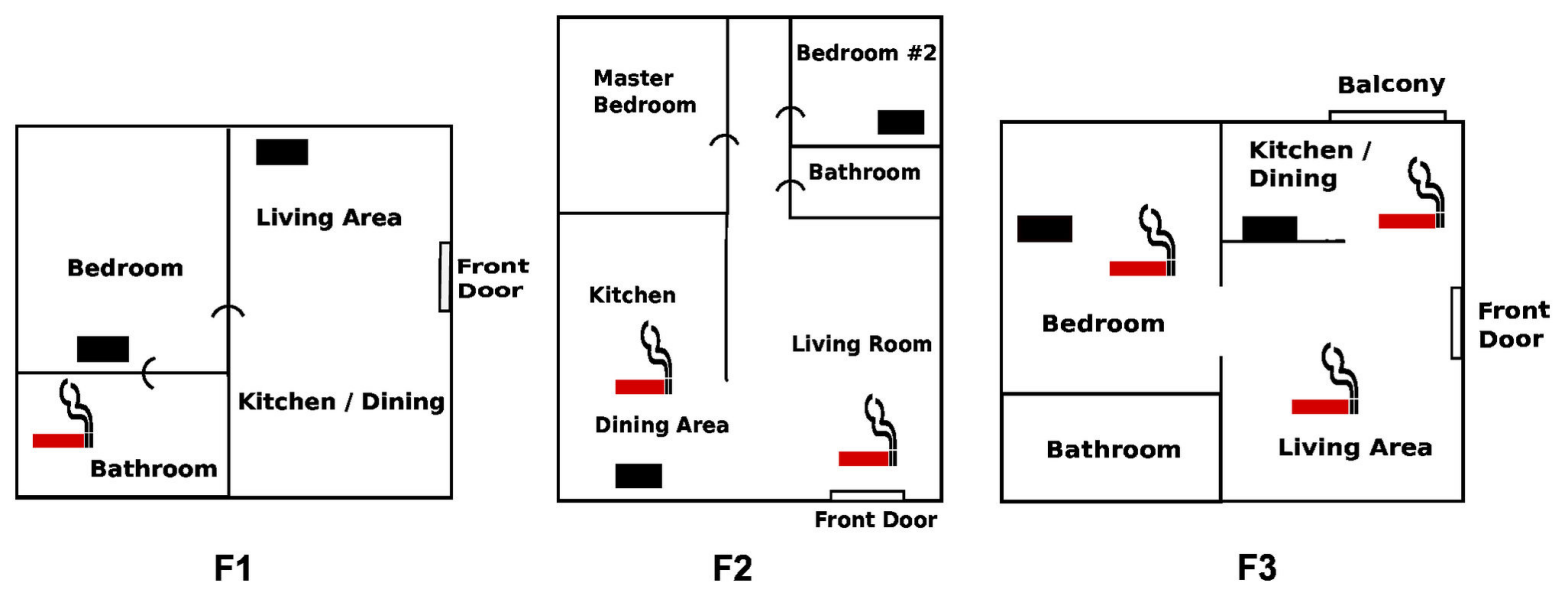
Room layouts for the homes of the three intervention participants (F1, F2, F3). Approximate reported smoking locations are designated with a red cigarette icon. Filled rectangles designate the location of the air monitors.

**Figure 4:**
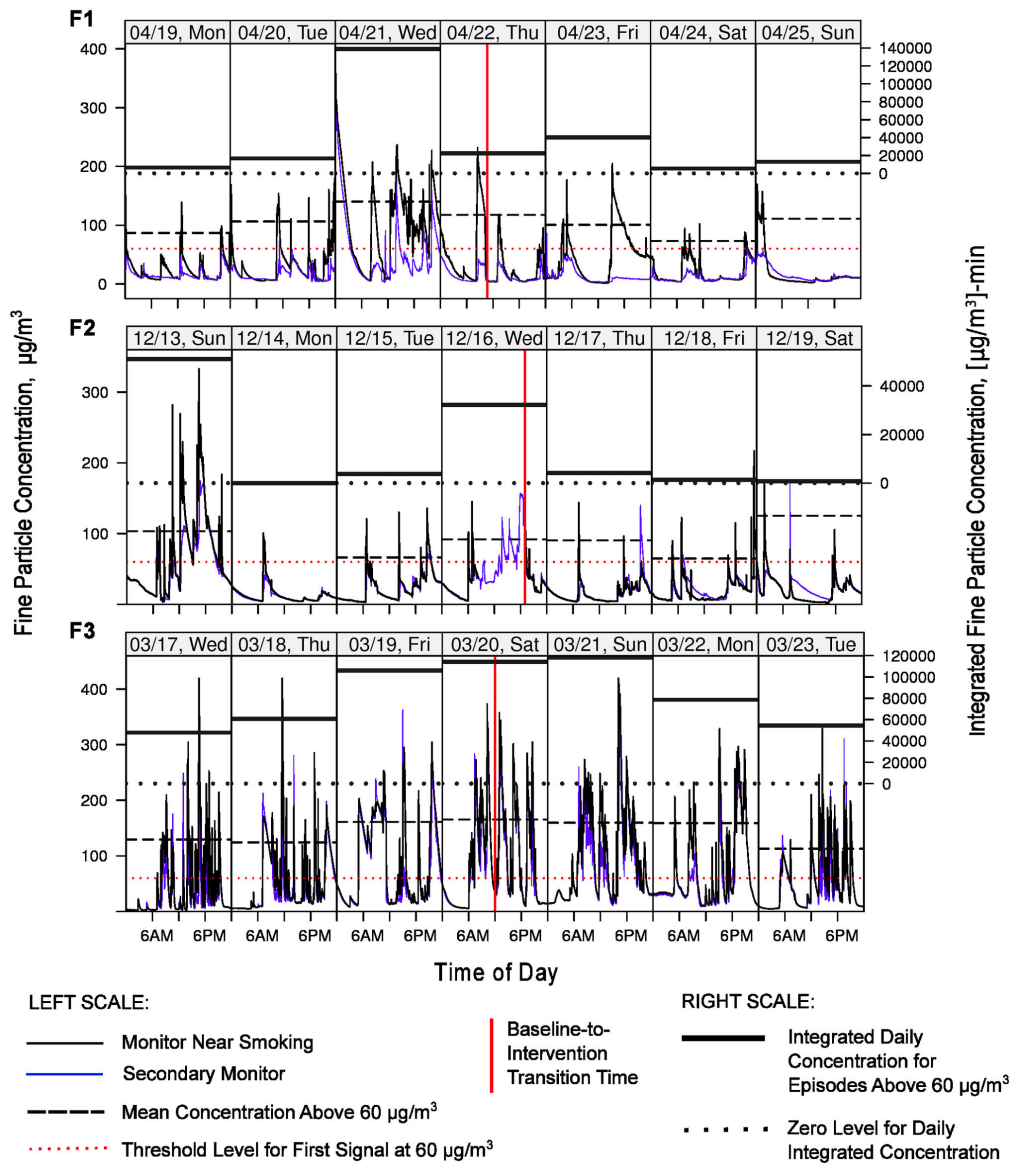
Week-long time series of 1-minute particle readings in the homes of the three intervention participants (F1, F2, F3). Data are shown for 7 days of monitoring near the primary smoking area (black line; Kitchen-Dining-Living area for families F2 and F3; Bedroom for F1) and a secondary area (thinner blue line; Bedroom for F2 and F3; Kitchen-Dining-Living for F1), starting with 3 days during the baseline period (before the signals were activated) and ending after 3 days of the intervention (after signals were activated). The middle day on each plot is the “transition day” on which the signals were activated and the intervention was initiated. The precise transition time is indicated by a vertical red solid line. Family F2 had missing data (due to monitor losing power) in the Kitchen-Dining-Living area for a large portion of the transition day, although both rooms track well for most periods, so the missing data can be inferred from the Bedroom results. The horizontal red dotted line designates the 60 µg/m^3^ first feedback-signal threshold (left scale). Black horizontal dashed lines designate the mean daily concentration of levels exceeding 60 µg/m^3^ in Bedroom for F1 and F2 and Kitchen-Dining-Living for F3 (left scale). Thick horizontal lines show the daily integrated (area-under-the-curve) values on the right scale in units of [µg/m^3^]-min for episodes when these same room levels exceeded 60 µg/m^3^.

The three families we studied (F1, F2, F3) varied substantially in their composition, baseline smoking behaviors, attitudes regarding SHS, and real-time particle profiles measured in a Bedroom and a broadly-defined Kitchen-Dining-Living area. Figure 3 presents simplified house layouts showing the position of each monitor and reported smoking locations. Each family responded differently to the in-home feedback, as reflected by interview responses and changes in the real-time particle measurements between the baseline and intervention periods. We systemically analyzed the particle levels observed in the three households by defining episodes (continuous time segments) during which levels continuously exceeded the first signal threshold of 60 µg/m^3^ (Table 3). By comparing the characteristics of the episodes before and after the signals were activated, we obtained a measure of the effectiveness of feedback in promoting SHS-reducing behavior. Table 3 provides a summary of episode-level statistics, broken out by family, monitoring area (Bedroom or Kitchen-Dining-Living area), and baseline or intervention period. The actual baseline and intervention time periods with full monitoring data covered smaller or larger periods than the 1-week target due to scheduling challenges or occasional equipment malfunctions. To illustrate the structure of the monitoring data, Figure 4 presents seven full days of time series data for each of the three cases, consisting of three days before the intervention started, a transition day, and three days afterward. Table 4 contains average daily counts of self-reported time-activity events occurring in the home for baseline and intervention periods: (1) Total cigarettes smoked per day; (2) Number of hours per day in which cooking and ventilation activity was reported to occur; and (3) Number of hours per day in which feedback signals was perceived.

**Table 3:** Smoking-Episode Metrics for Kitchen-Dining-Living Room (Kit-Din-Liv) and Bedroom Areas for Baseline (B) and Intervention (I) Periods for Participating Intervention Families (F1, F2, F3) ^**a**^.

Family ID	**F1**				**F2**				**F3**			
Monitored Area^b^	**Kit-Din-Liv**		Bedroom*		Kit-Din-Liv*		**Bedroom**		Kit-Din-Liv*		**Bedroom**	
Baseline or Intervention	**B**	**I**	**B**	**I**	**B**	**I**	**B**	**I**	**B**	**I**	**B**	**I**
Time Period, *Full Days* ^c^	6	3	6	3	4	11	4	11	6	5	6	5
No. Episodes, *Total*	25	2	53	15	18	86	10	34	102	81	72	90
No. Episodes *Per Day, Median*	4.5	1	9	6	6	7	3	2	18	16	12	15
Episode Duration *Per Day* (min), *Median*	213	3	379	154	98	72	57	46	571	495	481	481
Episode 1-min Peak Level (μg/m^3^), *Median*	74	75	96	88	111	102	68	74	114	125	108	78
Episode Average Level (μg/m^3^), *Median*	69	69	82	72	87	79	65	67	90	93	86	71
Episode Integrated Level *Per Day* (Conc x Time) ([μg/m^3^]-min), *Median*	19K	188	51K	13K	8.2K	6.6K	3.8K	4.2K	80K	80K	61K	55K

^a^Episodes are defined by continuous periods with particle levels exceeding the first signal threshold of 60 µg/m^3^ on full days of monitoring

^b^Areas (i.e., rooms) closest to regular smoking activity are indicated with an asterisk (*)

^c^We omitted transition days (i.e., days when signals were activated) in all statistics.

**Table 4:** Summary of the Average Number of Daily Time-Activity Events in Baseline (B) and Intervention (I) Periods for Participating Intervention Families (F1, F2, F3)^**a**^.

	**F1**		**F2**		**F3**	
	**B**	**I**	**B**	**I**	**B**	**I**
Cigarettes Smoked^b^	4.7	6.2	6.0	4.7	31	45
Cooking Events^c^	1.0	-	2.0	1.9	1.0	0.6
Exterior Window- or Door-Opening Events^c^	4.8	4.3	-	2.3	13	15
HVAC Events^c^	2.0	-	4	3.7	-	1.0
Marijuana-Smoking Events^c^	-	-	-	-	4.5	7.6
No. of Signals^c^,^d^	-	6.2	-	3.8	-	12.6

^a^The data in this table were obtained from participant self-reports. Participants filled out paper time-diary forms on a daily basis. Cells for which there were no reported data contain a hyphen "- " symbol.

^b^This row contains the average number of cigarettes smoked in the home per day, allowing for multiple cigarettes smoked per hour.

^c^These rows in the table contain the average number of hours per day that non-cigarette events were reported to occur. Multiple events within each hour were not considered for non-cigarette events, i.e., these data do not reflect more than one individual event that may have occurred within a given hour.

^d^The number of audible and/or visual signals that were perceived by participants were reported for the intervention (I) period.

### F1 Family Participant

#### Baseline Interview

Family F1 consisted of a man, a woman, and their 10-month-old baby. The interview was conducted with the woman. They lived in a 1-bedroom apartment (Figure 3). The kitchen had a screen door to the outside, and the bedroom and adjoining bathroom both had small windows. A fan was present in the bedroom, with a ceiling fan and radiator in the living room. When the participant was awake, the kitchen screen door was left open. The man (husband) was the only smoker and he smoked about 1 pack a day. He smoked in the bathroom with the door closed and window open (no fan), mainly at night and occasionally in the morning before he left for work or when he came home at his lunch break. Cooking was reported to occur for ~2 meals a day and there was a small vent fan in the kitchen wall. Cleaning made use of “pine sol, ajax, and bleach” and vacuuming, broom sweeping, and mopping. Floors were carpeted except for the bathroom and kitchen.

#### Post-Baseline Session

This session was conducted with the female participant who agreed to keep the monitors with activated signals for a week. During the scheduled visit, her husband was not available. During the visit, she was shown the chart of the particle levels for her home for the baseline period and discussed the probable source for the particles to be smoking, their risk to the family’s health, and what could be done to reduce the levels. She mentioned closing the bathroom door when her husband was smoking there at night, if he would not agree to smoke outside, since leaving the door open lets the smoke into the bedroom where all three family members sleep.

#### Post-Intervention Interview

This interview was conducted with the female participant until her husband joined the meeting when he returned home from work for lunch. She recalled hearing the feedback signals most days or everyday in the bedroom, but not in the living room. The signals occurred when the husband was home in mornings and evening or when he was home on weekends. They were able to see and hear the signals although they did not consider the sound to be loud. The feedback was not bothersome, but made them aware of the levels and that smoking led to harmful levels. They did not recommend any changes to the feedback signals. Being able to see or hear the signals served as a reminder, because they might forget to look at the chart. They liked the color of the moving chart so they could see the change in air quality level. In particular, the woman reported that her husband was surprised at how the feedback signals worked and he reported that it made him more aware, and he tried to go outside more and smoke less. The door to the bathroom was kept closed during smoking and windows opened to ventilate. The kitchen screen door was regularly kept open during occupancy as it was during baseline. The woman was surprised at how high the levels got when her husband was home and how low they were otherwise. She recognized the need to ventilate or smoke outside. She indicated that the monitors provided ‘constant’ reminders and that seeing the levels rise on the charts when someone smoked made them more aware of the polluting effects of smoking and led her to nudge the smoker to quit smoking or to smoke outside only. The woman said she would talk to her husband about a smoking ban in the house, although she usually leaves him alone to smoke when she is tired and goes to bed. When the husband arrived for lunch, the woman suggested that he quit smoking and they discussed the possibility of going to the doctor to get a prescription for him to stop smoking.

#### Time-Activities Diary

The woman completed the time-activity diary. The average number of daily smoking events appeared to increase somewhat from roughly 5 to 6 for the baseline versus intervention periods (Table 4). The number of cooking events (at 1-hour resolution) averaged one per day in baseline and decreased to zero during intervention. The frequency of 1-hour window or door-opening behavior was fairly stable at 4 to 5 per day.

#### Monitoring Data

We observed some changes in the SHS episode metrics between baseline and intervention (Table 3, Figure 4). Three days of monitoring were lost at the end of the intervention, leaving three full days of intervention data. The median number of episodes per day decreased by 78% in the Kitchen-Dining-Living area and 67% percent in the Bedroom, which was nearest to the smoking activity occurring in the adjoining bathroom. This effect indicates that indoor smoking was curbed. In addition, the median per-day episode duration decreased from over 200 to 3 minutes in the Kitchen-Dining-Living are and from 380 to 150 minutes in the Bedroom. Peak and average episodic levels were relatively stable, but the integrated levels had dramatic decreases of 99% and 75% in the two home areas. Figure 4 shows a decrease in the number of peaks and/or their height and duration just after the intervention started. While the number of smoking events may have remained the same or even increased slightly, the results indicate that behavior change in the home, perhaps in terms of moving smoking outside when signals were triggered, resulted in lower SHSe for occupants.

### F2 Family Participant

#### Baseline Interview

The F2 participants revealed positive attitudes on SHS reduction and smoking cessation during interviews and questionnaire responses. The F2 adult participants consisted of a female smoker who lived with her toddler and young dog, and her boyfriend who also smoked and visited her often. Scheduling visits was challenging, and the woman eventually participated in the study with a baseline period of 4 days. Cigarettes were generally smoked in the kitchen-dining area of the 2-bedroom apartment where one monitor was placed on a book shelf, with the front door occasionally open. The other monitor was placed on a shelf in an open closet in the child’s bedroom. The two adults reported smoking cigarettes roughly once per hour. Bedroom doors were generally left open. Cleaning activities included vacuuming the carpet throughout the living area and bedrooms, and degreasing the cooking area. Baking and frying were done in the kitchen and heating was provided by wall or portable heaters.

#### Post-Baseline Session

During the informal session, both smoking adults conveyed interest in the monitoring data and their worry concerning how long the smoke persisted after the cigarette was put out, and whether their neighbors were exposed. They were aware of smoking-related issues, with the discussion touching on the stigma associated with smoking, as well as the advent of many smoke-free policies in California. They both stated their intention to quit smoking, but needed more time, after having just purchased new packs of cigarettes, and that they perhaps needed some cessation-related assistance. The female adult did most or all of the indoor smoking. The boyfriend wanted her to smoke outdoors, away from the child, or to open a window. She was worried about being cold. The possible use of the kitchen exhaust fan, or blowing smoke out an open window, was mentioned. Both adults were enthusiastic about continuing participation in the study with the feedback signals turned on and viewing the graphical display. They agreed to the monitoring in their home for an extended intervention period of 11 full days over the December holidays.

#### Post-Intervention Interview

The post-intervention interview with the F2 female adult smoker indicated that she responded to the signals by trying to either open windows or smoke by the open door. She did not think that plots of data alone would have a lasting impact, but the signals made her want to improve the air quality by smoking outdoors or by quitting. She indicated that she and her boyfriend wanted to quit smoking and thought that keeping the monitor would help reduce smoking or aid them in quitting.

#### Time-Activities Diary

The woman completed the time-activity diary on a daily basis. The number of reported cigarette, cooking, and HVAC events per day were roughly consistent between the baseline and intervention periods (Table 4). However, more than 2 window or door-opening hourly events per day were reported during the intervention, and none were reported during the baseline period. The reported average of 4 feedback signal events experienced per day (at 1-hour resolution) appeared to underestimate the number of expected signals derived from monitoring data (Table 3).

#### Monitoring Data

**Figure 5:**
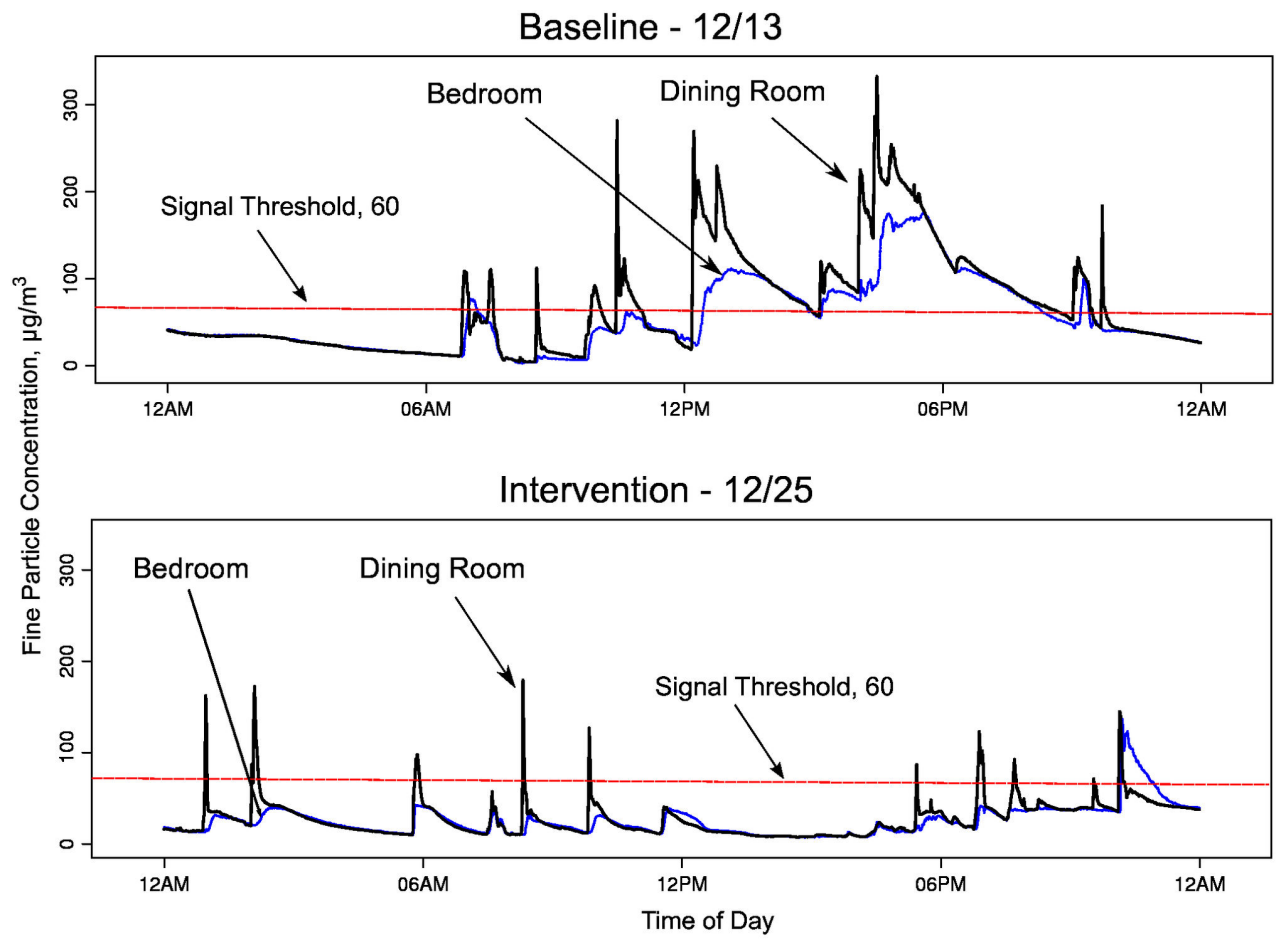
Day-long 1-minute particle levels in home F2 for selected days during baseline and intervention. The black line designates the Kitchen-Dining-Living area monitor readings (monitor placed in Dining Room) and the thin blue line designates the Bedroom monitor readings (child’s room).

The reported behavior changes between the baseline and intervention periods are evident in the physical monitoring data (Table 3, Figures 4 & 5). Four full days of baseline and 11 full days of intervention data are available. While the median number of episodes per day changed by 1 occurrence, there were drops of 26 and 11 minutes in the median daily duration of episodes (98 to 72 minutes in the Kitchen-Dining-Living area; 57 to 46 min in the Bedroom). The median per-episode peak and average levels in the Kitchen-Dining-Living area dropped by a smaller proportion, and increased slightly in the bedroom. Integrated levels (area under the curve) dropped by 20% in Kitchen-Dining-Living and increased by 11% in the Bedroom. Figure 4 presents the integrated daily metric, which reflects both the per-day concentration and duration of the episodes. Integrated levels decrease as peaks become sharper (shorter source duration). Overall, the decrease in main living area per-episode cumulative levels provides evidence that the behavior of the smoker changed. The decrease in the higher absolute levels in the Kitchen-Dining-Living area outweighed the marginal increase or stability in the lower absolute levels in the bedroom, likely resulting in decreased cumulative exposure for the child and other occupants.

To provide a more detailed illustrative example of changes in time series features, Figure 5 shows daily real-time levels observed for a single baseline day and a single intervention day. Hard copy versions of similar plots were shown to study participants after the baseline and intervention periods to provide additional intervention-related feedback, providing visualization of the degree, timing, and persistence of SHS levels and their spread throughout the home. The time series are distinguished by sharp peaks in particle levels in the Kitchen-Dining-Living area, presumably because the female smoker in the house typically lit up near this monitor, followed by gradual decreases in levels. During the intervention period, the signals were first triggered when levels exceeded a threshold of 60 µg/m^3^. The participant reported moving to an open door to smoke or opening a window in attempts to lower the levels. For the intervention day shown, the levels appear to drop below the threshold more rapidly than on the baseline day. In this case, the peak levels are generally lower during intervention, although this effect may not have occurred all the time. The levels in the bedroom did not typically have large transient peaks that might arise from proximate smoking, but rather they reflected the rapid mixing of particles in the small apartment. Within approximately 1 hour after the start of a smoking episode, and prior to another smoking episode, levels in the Kitchen-Dining-Living area and the Bedroom were similar in magnitude.

### F3 Family Participant

#### Baseline Interview

This family consisted of a man, his pregnant wife, his father, and a young dog. The family lived in a small 1-bedroom apartment with a wide double-door dividing the bedroom from the living area, which was usually kept open (Figure 3). The apartment had one window in the bedroom and a balcony door near the kitchen, which was usually open during the summer. Both the man and his father regularly smoked menthol cigarettes in the home throughout the day, and the man also smoked medical marijuana in the home on a regular basis. Smoking was allowed in all rooms with most smoking occurring in the living room on the couch near the television. There were wall heaters, which were generally not used. During smoking, the kitchen fan was turned on and patio door opened. Cooking was performed regularly in the home using the microwave and electric stove. Cleaning was done using powder cleaner with vacuuming, and sweeping with a broom. The kitchen had linoleum and the rest of the apartment had low-pile carpet.

#### Post-Baseline Session

All three family members agreed to have the feedback signals activated for a 1-week period. They were showed their time series plots of the particle levels from the baseline period and they identified the sources of the peaks. They were told that the goal was to lower their particle levels as much as they could for their family’s health. They discussed several potential mitigating actions, e.g., turning on the stove vent, airing out the room, and smoking outside. When smoking outside was mentioned, the man and his father joked about the “baby being tough” like them and able to handle the smoke; the woman was quiet during that comment. The men in the family were not positive about the possibility of changing their smoking behavior.

#### Post-Intervention Interview

The family reported seeing the red light “all the time” and hearing the signals around 5 or 6 times a day, especially on the weekends and around 3 PM when the man came home from work. They did not report any preferences for changing the signal sound or lights and they did not find them frustrating or annoying. They did not have any suggestions for other uses of the monitor or making it more effective. They reported smoking as much as before, but they would point the kitchen fan at the monitor and opened the patio door, and the father had shifted to smoking inside by the patio door. When they barbequed on the balcony, they kept the door closed. They felt that the signals helped them to get the levels lower in the home. The woman stated that the chart of the levels that was presented to the family made her “feel sad to know that the air is bad and unhealthy.” After that statement, the smokers said that they were willing to consider smoking outside or quitting smoking to reduce smoke levels further, but that no changes in smoking would occur before the baby was born. The man said he would quit smoking after the baby was born; he was not receptive to smoking outside, but his father was open to it. The family joked that they would “move [the] couch outside” since that was where the man liked to smoke. The family was given smoking cessation materials.

#### Time-Activities Diary

The woman completed the time-activity diary. There was an apparent increase in reported daily mean number of cigarette events between the baseline or intervention periods (Table 4) with 31 and 45 cigarettes smoked per day on average for the baseline and intervention periods, respectively. Marijuana-smoking activity also apparently increased. The level of cooking and window/door-opening activity appeared similar in the two periods. During the intervention, the signals were reported to trigger about 13 times a day at 1-hour resolution, although more than one cigarette may have been active in a given hour. The HVAC system was reportedly used, on average, once per day in the intervention but never used in the baseline period.

#### Monitoring Data

We did not observe a decrease in SHS particle levels for the main smoking room (Kitchen-Dining-Living monitor) between the baseline and intervention periods, but there may have been a small decrease in the Bedroom area (Table 3, Figure 4). Six full days of baseline data and 5 full days of intervention data are available. The median number of per-day episodes above the first signal threshold remained fairly stable, as did the median per-day episode duration. While the median peak, average, and daily integrated levels for episodes were stable or increased for the Kitchen-Dining-Living area, they decreased by 10% to 28% for the Bedroom, possibly a result of the position of smoking in the home (e.g., near the patio) or the use of fans, the bedroom door position, or perhaps use of the HVAC. However, the absolute SHS levels remained very high in both areas of the home. Figure 4 shows consistent and high particle levels without a clear trend in number or sharpness of peaks. These results are consistent with self-reports that smoking activity did not abate or move outdoors in response to signals, but subtle movement in smoking-related behavior may have occurred.

## Discussion

### SHS Sensitivity and Specificity

The Dylos monitor has adequate sensitivity to tobacco smoke and adequate accuracy in indicating elevated particle mass concentration levels from smoking events. We established a linear relationship between the Dylos and a mass-measurement device (mass-calibrated DustTrak, TSI, Inc.) over the range of feedback signal thresholds of 60 to 100 µg/m^3^, which were selected to respond to smoking events in homes. Another research team [31] studied the relationship of the Dylos against a similar device (Sidepak, TSI, Inc.), finding a non-linear relationship that becomes pronounced above 100 to 200 µg/m^3^. Their findings appear roughly consistent with our results showing a mass-concentration versus Dylos relationship that can be treated as approximately linear for lower levels. Our findings regarding the Dylos’ sensitivity and linear relationship to gravimetric particle measurements are consistent with the non-tobacco work of Northcross et al. [32], who evaluated a customized device essentially equivalent to those used in the present research. However, for levels above approximately 150 µg/m^3^, our linear conversion equation may under-predict the true particle mass concentration.

Like all real-time particle measurement devices, the response of the Dylos is not exclusive to a given particle source, so rapid feedback cannot be easily focused on tobacco smoke events exclusively. For example, the Dylos responds to cooking emissions, incense, and other combustion sources. However, with knowledge of time-activities in a home, it appears possible to identify peaks from smoking with good accuracy. Also, house occupants are likely aware of the different particle sources active at any given time and, with adequate instruction, may be able to discriminate the source or sources associated with real-time feedback. In the future, sensor technologies with better tobacco specificity may become available, e.g., real-time nicotine sensors, or perhaps sensor combinations will be feasible. Information on the particle size distribution alone may also be used to identify sources, e.g., by incorporating the size bins measured by the Dylos device to discriminate sources by establishing characteristic size distributions for different classes of sources. Dacunto et al. [33,34] report that the Dylos may be a useful tool for identifying different particle sources in single-room or multi-unit-housing settings. The present system broadly differentiates sources by filtering out particles above 2.5 microns that may be associated with dust rather than tobacco smoke. However, being able to provide real-time particle feedback that adjusts automatically to different sources is complex and requires further research and sensor development.

### Acceptability and Feasibility

We are unaware of any prior studies that have deployed a real-time SHS feedback system in the homes of smokers, although Kim and Paulos [35] describe residential evaluation of a real-time tool for general indoor air quality education (not source specific) consisting of a Dylos and mobile phone. Wilson et al. [12] found that providing particle feedback to smoking mothers was feasible and acceptable, but the feedback was not provided in real time. Kim and Paulos [35] report general feasibility, increases in awareness, and some behavior change for home occupants, but also frustration with what the levels meant in terms of particle source and how occupants should address them.

For our work, the convergence of inexpensive and reliable components, including SHS sensors, customizable microchips with sounds and lights, and standard wireless and internet communications, have made it possible to consider a flexible, practical and affordable in-home system giving immediate feedback on SHS levels. We determined that such systems would be welcome in households with interest in improving their home’s air quality. Once installed, the units can notify occupants of increasing levels in an acceptable manner that may lead them to modify their behavior immediately and over time. However, the actual impact of long-term deployment has not been evaluated. Our feedback configuration -- in the form of simple lights, sounds, or scrolling data visualization – appeared to be well received, although various improvements were identified. Based on feedback from study participants, we may explore user-visible improvements to the monitoring and feedback system including: (1) Smart signals that change characteristics for sustained SMS levels versus transient elevation; (2) Enhanced on-device signal features, including more pleasing sounds (e.g., music, speech), and LCD display of real-time levels and images showing severity of pollutants; and (3) Integration with web devices or smart phones to facilitate email communications, use with social media, and provide tips and information on SHS and mitigation measures. Improvements that would streamline data management and formulation of feedback in research include: (1) Fine-tuning of wireless transmission and reception of data into a web application that serves historical data to study investigators or participants; (2) On-device controls (with password protection) for changing signal settings; and (3) An integrated SD card or USB interface for easy on-site data download from devices.

### Impact of the Intervention in Familial Context

The clinical impact of the intervention is rooted in the familial contexts in which it is applied. Rule-governed behavior, which our intervention seeks to establish, is expected to depend on the initial parameters at the first contact with the family, specific instructions given to different family members, and relationships within the family. For our three intervention families, the starting points of the intervention were quite different, ranging from a one-smoker family already engaged in what was perceived by the family as mitigating behavior (i.e. smoking in a closed bathroom with the window open), to an informal two-smoker couple with existing cessation sentiments, and, finally, to a two-smoker family with one smoker having a hard stance to continue smoking as usual and the other with a potentially softer attitude. In each case, we informed the participants of possible ways to mitigate the SHS in the home, should they choose to do so, but we did not instruct them to take specific action. Each family found its own way to respond to the light, sound, and graphical feedback signals. By viewing the graphical real-time display, they were able to immediately see the result of specific behaviors. The first and second families appeared relatively receptive to the feedback at the outset and continual information on the degree of contamination seemed to increase their awareness, prompting them to take further action to reduce SHSe for their children and/or themselves, such as smoking outside, smoking less, or smoking near a doorway or window. The monitoring data and self-reports indicate that SHSe was reduced for the child and others in the home. In contrast, the third family initially expressed a clear non-receptivity to any changes in smoking behavior, perhaps influenced by a single strong personality. Also, this family did not yet have a child in the home, so the smokers’ motivation may have been lower. The monitoring data reflected this unwillingness to change with the maintenance of very high SHS exposures for all occupants, although perhaps with a small decrease in bedroom levels. However, at the end of the short 1-week intervention some members of the family voiced concern about the level of smoke or, as a smoker, raised the option to smoke outside when the baby was born. In this case, the intervention may have provided a catalyst and objective support to begin tackling a previously-unaddressed worry about SHSe.

### Development of Real-Time Behavior Feedback

Counseling-only interventions have been effective in some studies, with predominantly modest effects [9,36]. Such modest effects may be due to limited affordable counseling time, the inability to provide understandable and credible feedback on the level of smoke exposure in the home, or the inability to contextualize specific smoking practices and their relationship to SHS levels. In Hovell et al. [36] various levels of prompting and positive verbal feedback were provided for gradual reductions in children’s exposure to SHS. Presumably, social contingencies of reinforcement from family and friends contribute to the reduction in SHSe or the failure to attain such a reduction. However, counselors were not present to directly promote social reinforcement for SHSe reduction in the home. Thus, the counseling process was incomplete and operated on limited and delayed data about smoking, SHSe, and social contexts.

Previous intervention studies that employed delayed cotinine or air nicotine feedback indicated that SHS exposure occurred but provided limited information on how or when they occurred, suffered from similar limitations as counseling interventions, and showed mixed results [37,38]. The recent Wilson et al. [12] pilot study, which combined delayed counseling and delayed SHS particle feedback in the form of charts, did not provide real-time reinforcement or shaping. But its use of minute-by-minute particle data on printed charts appears to have enabled participants to understand the severity of SHS levels, to interpret the impact of their original actions, and to take new action to reduce levels. Wilson et al. [12] observed significant reductions in particle levels due to the intervention, as well as “shock and surprise” on the part of study participants regarding SHS levels, and increased motivation to change smoking behavior, especially to protect their children’s health.

In contrast to delayed-feedback studies, even ones that include time series charts as feedback, real-time interventions like the present study provide immediate contextual information on the degree and timing of the SHS hazard, likely allowing occupants to more easily explore tailored mitigation options and observe immediate impacts. Dynamic graphical feedback with short-term history, provided on a computer display, appears likely to provide the most easily-interpreted information to help family members visualize how their actions lead to specific SHSe increases or decreases.

The results of this pilot study, which establish positive potential for a real-time system to bring about better health practice, are likely to support a host of new SHSe studies incorporating real-time and repeated measures to shape behavior over a longer intervention period. Tested tobacco and SHSe control measures, which are based on real-time behavior theory such as the BEM, may eventually become acceptable on a large scale in housing, vehicles, workplaces, or motels and hotels to disclose contamination and protect the public and vulnerable persons (e.g., children, the elderly, and persons with asthma). The technological costs of the intervention do not seem prohibitive relative to consumer items currently owned by low-income families (cell phones, TV’s), and may decrease as more technologies come to market. In particular, smart phones, which are becoming commonplace in low-income and other households, might be combined with future low-cost sensors to provide affordable and convenient feedback.

An important future advancement in the SHSe feedback, and efforts to test hypotheses from real-time behavioral theory, will be the ability to automatically modify feedback parameters (e.g., threshold values, sound, volume, light intensity) following detected changes in behavior or SHSe levels. Adaptive interventions, which employed *auto-shaping* feedback daily, have recently been used for physical activity [39]. A similar auto-shaping capability, when applied in real-time to SHS interventions, would allow the feedback device to follow a programmed pattern (i.e., rules) of feedback modification designed to achieve and maintain reduced or eliminated SHSe.

According to the BEM, the dynamics of SHSe and smoking behavior in homes are likely influenced by social contingencies. These may include whether or not visitors are present, and which family members are home at key moments in time. For example, if smoking guests are present, they may be less likely to be asked to go near the door to smoke. Or, perhaps one’s sister is more adamant about not smoking in the home than other family members. Future studies should formulate and test hypotheses concerning a range of social contexts and pressures that can occur in a home to make an intervention more or less successful. In addition, multiple intervention strategies may be employed simultaneously, including real-time feedback, counseling, and cessation-focused approaches.

The BEM [13,14], which formed the foundation for the present intervention, may prove to be a successful framework to formulate hypotheses and drive real-time behavioral science. Riley et al. [15] recently noted that traditional behavior theory needs an overhaul to support emerging mobile health interventions, perhaps with a systems engineering control approach to theorizing moment-to-moment behavior, as incorporated by the BEM theory. Popular concepts of “feedback loops” [40], such as providing dynamic traffic speed signs, where immediate environmental and social stimuli work to influence behavior, also have common roots in the BEM.

## Conclusions

Based on lab tests, focus groups, field tests and field interviews, the monitoring system we developed in this study provides a feasible, acceptable, accurate, and practical means to provide feedback to household residents on the real-time level of SHS-related pollutants in their home. The system showed potential for modifying smoking behavior in homes and reducing SHSe levels using audio-visual and graphical feedback. Graphical representations of real-time levels, in particular, may provide compelling feedback for families to pursue mitigating approaches to reduce SHSe and eventually implement a home smoking ban. Future research using controlled trials and larger sample sizes should deploy and evaluate a refined system in smoking homes, and other locations with smokers, such as vehicles or multi-unit housing. Studies should examine the impact of the monitor-based intervention in terms of both short and long-term SHSe reduction as compared to other tobacco control approaches, e.g., counseling-only. Permanent installation is a possibility that may result in stable behavior change. Small samples of participants may be justified, especially if detailed information on subject time-activities and social contexts can be measured coincidentally with SHSe. The system might also be used to study feedback on aerosol levels from various non-tobacco sources and asthma triggers. Future refinements to the system, such as on-device panel controls or LCD display, better-tailored lights and sounds, more options for visual and graphical feedback, and more convenient data-recovery options should improve the system’s effectiveness and ease-of-use.
